# Supplementary material for: A Critical View of the Application of the APEX Software (Aqueous Photochemistry of Environmentally-Occurring Xenobiotics) to Predict Photoreaction Kinetics in Surface Freshwaters
Source: Molecules. 2019 Dec 18;25(1):9. doi: 10.3390/molecules25010009 (PMC7017383; doi:10.3390/molecules25010009)
Supplement: Supplementary file 1 [file molecules-25-00009-s001.zip › 3.2.4_gcc-4.4.0.zip!/3.2.4_gcc-4.4.0/3.2.4_gcc-4.4.0/doc/octave/html/interpreter/System-Utilities.html]

System Utilities - Untitled


Next: Packages,
Previous: Object Oriented Programming,
Up: Top


---

## 34 System Utilities

This chapter describes the functions that are available to allow you to
get information about what is happening outside of Octave, while it is
still running, and use this information in your program. For example,
you can get information about environment variables, the current time,
and even start other programs from the Octave prompt.

- Timing Utilities- Filesystem Utilities- File Archiving Utilities- Networking Utilities- Controlling Subprocesses- Process ID Information- Environment Variables- Current Working Directory- Password Database Functions- Group Database Functions- System Information- Hashing Functions
